# Outcomes assessment pitfalls: challenges to quantifying knowledge gain in a sex education game

**DOI:** 10.12688/gatesopenres.13129.3

**Published:** 2021-02-16

**Authors:** Elena Bertozzi, Amelia Bertozzi-Villa, Swathi Padankatti, Aparna Sridhar

**Affiliations:** 1 Game Design & Development, Quinnipiac University, Hamden, Connecticut, 06518, USA; 2 Institute for Disease Modeling, Bellevue, Washington, 98005, USA; 3 Department of Pediatrics and Neonatology, Sundaram Medical Foundation, Chennai, India; 4 Department of Obstetrics and Gynecology, David Geffen School of Medicine, Los Angeles, CA, 90095, USA

**Keywords:** games for health, serious games, sexual education, outcomes assessment, family planning, India

## Abstract

**Background:** The use of videogames as a public health tool is rapidly expanding. Accurate assessment of the efficacy of such games is complicated by many factors. We describe challenges associated with measuring the impact of playing a videogame with information about human sexual anatomy and reproduction and discuss motivations for, and solutions to, these challenges.

**Methods**: The My Future Family Game (MFF) is a validated tool for collecting data about family planning intentions which includes information about human anatomy and sexual reproduction. We sought to assess the efficacy of the game as a tool for teaching sexual education using a pre-post model which was deployed in three schools in and around Chennai, India in summer of 2018.

**Results:** The MFF game was successfully modified to collect data about players’ pre-gameplay knowledge of sexual anatomy and processes. The post gameplay assessment process we used did not effectively assess knowledge gain. Designing assessments for games dealing with sexuality presents challenges including: effectively communicating about biological parts and processes, designing usable and intuitive interfaces with minimal text, ensuring that all parts of the process are fun, and integrating assessments into the game in a way that makes them invisible.

**Conclusion:** Games can be an effective means of gathering data about knowledge of sex and reproduction that it is difficult to obtain through other means. Assessing knowledge about human sexual reproduction is complicated by cultural norms and taboos, and technical hurdles which can be addressed through careful design. This study adds to the sparse literature in the field by providing information about pitfalls to avoid and best practices in this evolving area.

## Introduction

The acceptance of games as useful and effective tools for collecting data, educating players, and achieving positive behavior change is growing due to an increase in rigor in the deployment and assessment of serious games ( Boyle *et al.*, 2016; Coovert *et al.*, 2017; Zammitto, 2009). Embedding outcomes assessment within the game itself is often described as an important design principle in building games, largely due to the fact that most games incorporate some form of player feedback and metrics as part of gameplay ( Ifenthaler *et al.*, 2012; Van Staalduinen & de Freitas, 2011). There are situations, however, in which such assessment is quite difficult.

The *My Future Family Game* (MFF) game was initially developed as a tool for collecting information about family planning intentions among adolescents in Mysore, India in 2017. The original goal was to gather information about desired family size and spacing, influencers of the decision-making process, and other data points ^i^. Focus group participant feedback during early-stage planning was crucial to the success of the project. Serious games are only effective if in addition to achieving their stated goals, they are also intrinsically motivating (fun) for players ( Malone, 1981). Analysis of focus group feedback showed that although sex education is included in the standard curriculum for adolescents, many young people do not have basic knowledge about human reproduction ( Bertozzi *et al.*, 2018). Including this information in the game would strongly motivate adolescents to play, and was supported by parents and educators as a way of communicating sensitive information.

The first beta of the game (MFF_2017) was successfully tested on 480 adolescents in summer of 2017 and proved to be a very effective tool for gathering information from a population about which little accurate information is available from other sources ( Ismail *et al.*, 2015; Weiser, 2015).

Post-game paper questionnaires and interviews demonstrated that the game was considered fun and well-accepted by student players. Analysis of the MFF_2017 deployment suggested that the game could function not only as a method of collecting data about family planning intentions, but also as a means of communicating information about human sexual anatomy and reproduction in an innovative way ( Bertozzi *et al.*, 2018). For the second deployment of the game (MFF_2018) on a different population we sought to validate the utility of the game as a means of measuring pre-intervention knowledge of game content and quantifying knowledge gain after having played the game. Our goal was also to develop an assessment that did not require post-intervention interviews or paper questionnaires to facilitate large-scale deployment of the game as an educational tool in low-resource settings.

A literature search was conducted to assess best practices for outcomes assessment of videogame efficacy particularly relating to games for health and knowledge gain. There is very sparse literature in the area and several of the papers discussed this lack and the need for more work in the field ( Baranowski *et al.*, 2019; DeSmet *et al.*, 2015). The search terms ‘videogame’ AND ‘knowledge’ were used in the PubMed database resulting in 38 results. Titles and abstracts were screened for studies that measured outcomes of playing videogames about health resulting in 16 papers that discussed methodologies. Of these, papers that reported assessment of knowledge gain as a result of videogame play dealt with topics that either were integrated with existing assessments (such as diabetes knowledge measures ( Joubert *et al.*, 2016)) and/or were not controversial and easily assessed with pre-post questionnaires ( Del Blanco *et al.*, 2017; Hieftje *et al.*, 2019).

 A search using ‘videogame’ AND ‘sex yielded 51 non duplicative results of which only two were assessments of a videogame about sex from the same research group ( Fiellin *et al.*, 2017; Gariepy *et al.*, 2018). Neither of these attempted a pre-post model for knowledge gain. The primary outcome measure was behavioral (initiation of sexual activity). Players of the sex ed game demonstrated increased sexual knowledge (assessed using interviews) compared to a control group that played generic games.

The literature provided us with little guidance in determining how to assess knowledge gain in a videogame about sexual education that did not include in-person interviews, beyond the importance of embedding the assessment as much as possible within the game itself.

## Methods

### Inclusion Criteria and Deployment Structure

MFF_2018 was deployed in Chennai, India as part of research conducted by Dr. Swathi Padankatti and her team from the International Alliance for the prevention of AIDS in collaboration with the U.S. based game development team (Dr. Bertozzi’s group at Quinnipiac University) and Dr. Aparna Sridhar at U.C.L.A’s School of Medicine. Dr. Padankatti and her team identified three schools willing to participate in the study who could provide a total of 419 student players (208 males and 211 female) ranging from 13 to 16 years of age. This was a convenience sample due to the sensitive nature of the game content. Schools were selected based on the research team’s pre-existing relationships with administrators, with whom they had previously worked on AIDS education initiatives and were open to curriculum initiatives relating to sexual knowledge. The fact that these educators and students had previously participated in HIV education initiatives made it likely that the sample audience would have more knowledge about sex and reproduction than the population at large.

For game deployment in each participating school, 30 android tablets and headsets were set up in a school classroom, and groups of students in the target age group successively cycled through to play the game and discuss their experience. Participants were invited to play the game if they desired to do so and could stop at any time. No students were excluded from participation. Groups were not segregated by sex. To ensure comfort and privacy, students were able to move freely around the room to find their preferred space to sit and play.

In the MFF_2017 deployment post-game questionnaires were used for assessment. These were paper forms filled out by students after playing the game, asking students to qualitatively self-assess knowledge gain and provide feedback on the process of gameplay. While these questionnaires provided valuable feedback and indicated high rates of self-assessed knowledge gain, they were not efficient data-collection strategies. Because forms were filled out on paper, response rates were low and it was not possible to link student feedback to specific test-takers. Positive self-assessment of knowledge gain was encouraging, but not a rigorous method for determining game efficacy. The absence of an evaluative framework for the game was the primary motivation for development of the pre-post testing process in MFF_2018.

Once the participants began playing, they were first asked to indicate their sex and age, after which the pre-test was triggered prior to initiating the main game. The post-test, with exactly the same structure and questions as the pre-test, appeared after completing the game.

Given that MFF_2018 was the first field deployment of the pre-post assessment, the deployment team reported issues to the U.S. development team after each play session. The issues were collected and organized into topics. Below we describe the challenges encountered in both the MFF_2017 and MFF_2018 tests and how they were addressed or are planned to be addressed in future revisions of the game.

*MFF (original and modified versions of the game are available here:* https://osf.io/gtfu5/wiki/home/ ( Bertozzi-Villa *et al.*, 2020). The apks can be installed on any Android tablet or phone.). A video of gameplay can be viewed here: https://familyplanninggame.qu.edu/ which includes all the minigames.

### Pretest Challenge One: Communication

Discussion of human anatomy and behavior regarding sex and reproduction is problematic in India ( Ismail *et al.*, 2015). Many adolescents receive very little information from their parents or teachers due to cultural taboos ( Khubchandani *et al.*, 2014). In designing the MFF_2017 game, we were very careful to introduce explicit material slowly and through a process in which it was revealed in context. The game was constructed so that at each point where explicit material is available for the player, the player was asked whether or not they wanted to see it, and then if they agreed, the material was presented in a context that made sense based on the information being gathered.

**Figure 1.**
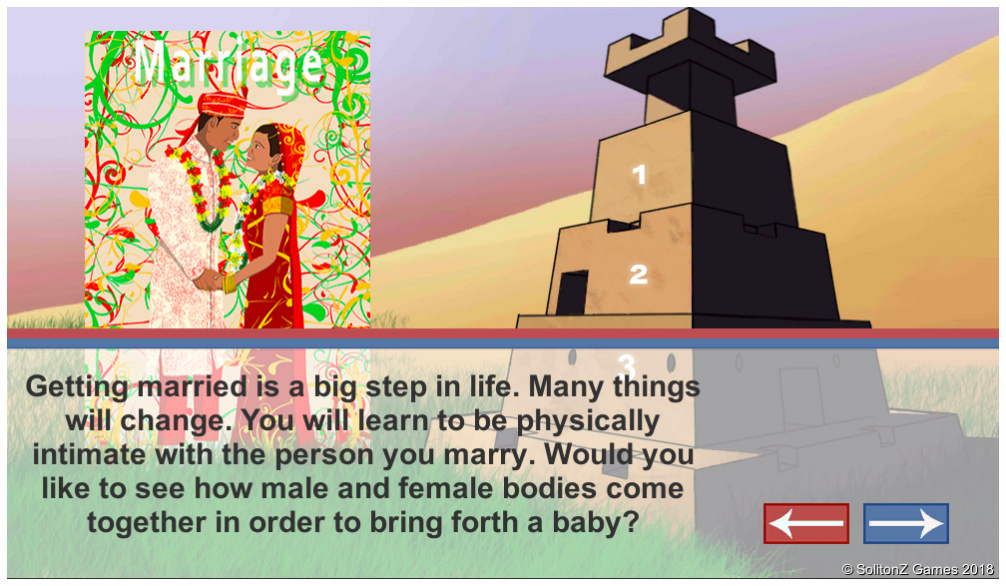
Consent screen from Getting Married Milestone.

**Figure 2.**
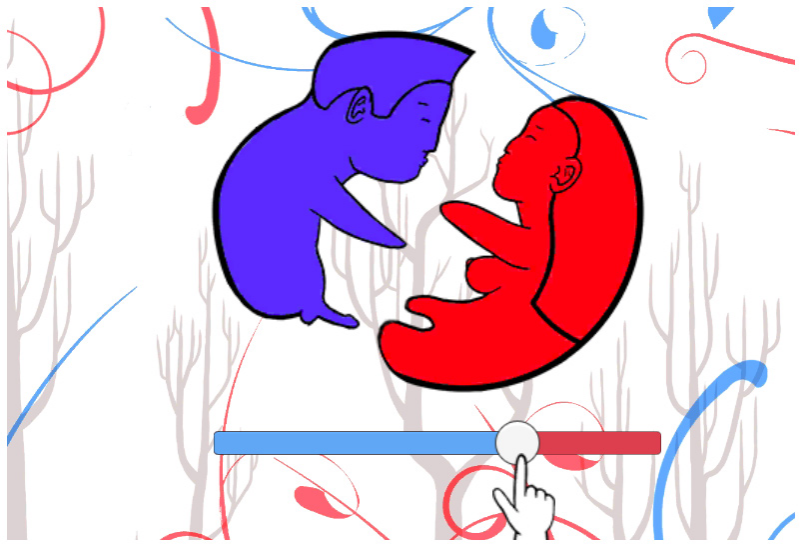
Still from animation that demonstrates sexual intercourse.

For example, when players were asked information about when they planned to start dating a possible partner, they were provided with information about the anatomy of the opposite sex ^ii^. When they were asked about the age they planned to marry, after consenting ( Figure 1), they were given information about how intercourse works via the animation in Figure 2.

**Figure 3.**
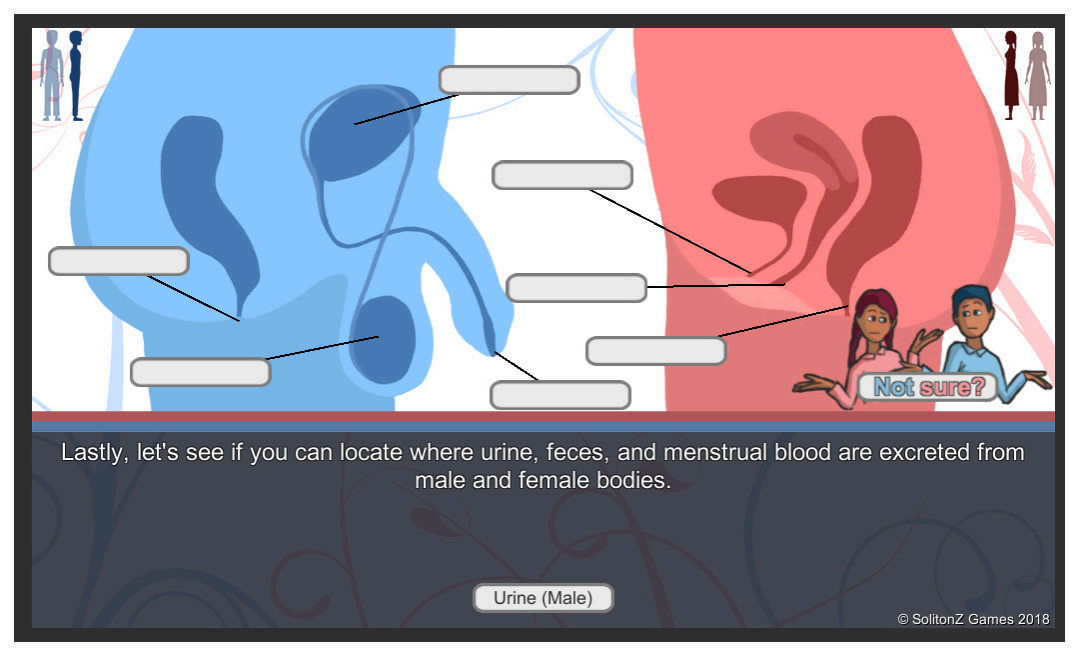
Final anatomy screen from pre-test.

The addition of a pre-test that collects knowledge of human sexual anatomy and reproduction before the game begins required exposing participants to images and terms before we were able to prepare them the way we had with gameplay without assessment. To be as accessible as possible to players at any reading comprehension level, the game includes as little text as possible and communicates most information through graphics, audio and animation. This is especially important when discussing information about sexuality because these terms may not be familiar to students. However, the inclusion of the pre-test introduced a great deal of technical vocabulary in English before the gameplay began. All of the schools included in the MFF_2018 study had instruction in English, but it was unclear if terms like testicles, ovaries, urine and feces were well-understood by players ( Figure 3). Although education about sexual functions is technically part of the educational curriculum for all students in India, the content is not actually taught in many schools due to cultural reluctance to discuss sexuality.

Solution: We sought to address the challenge of not startling participants by designing an interface that introduces the sensitive content in the pre-game assessment carefully. Images are largely outlines with enough detail to communicate but not enough to be offensive. Biologic terms relating to specific body parts are shown in limited number on the screen where they belong so even if the player does not know exactly what the term means they can start to associate the correct term with the appropriate body parts. Players are taught the correct terms and their associated body parts when they play the game.

### Pretest Challenge Two: Interface Design

A usability issue encountered during the MFF_2017 was that players lacked familiarity with the drag and drop interface commonly used on smartphones and tablets. The design team determined that the 2018 pre-test was the perfect opportunity to teach players how to use drag and drop so that they would be prepared for it when they reached the game. We also determined that we could use the interface design to reinforce the learning of anatomy by clarifying the correlation between the body parts pictured on the screen and their own bodies as explained below.

The key educational content of each milestone of the game is outlined in Table 1.

**Table 1.** Key educational content for each milestone in the MFF game.

Milestone	Content
1: Puberty and bodily functions (same sex as player)	Hair growth, menstruation or ejaculation
2: Reproductive anatomy	Identification of the internal reproductive organs of male and female bodies and their functions
3: Puberty and bodily functions (opposite sex as player)	Hair growth, menstruation or ejaculation
4. Anatomy of intercourse	Act of heterosexual coitus via union of penis and vagina
5. Fertilization	Movements of eggs and sperm, fertilization of egg by sperm.

**Figure 4.**
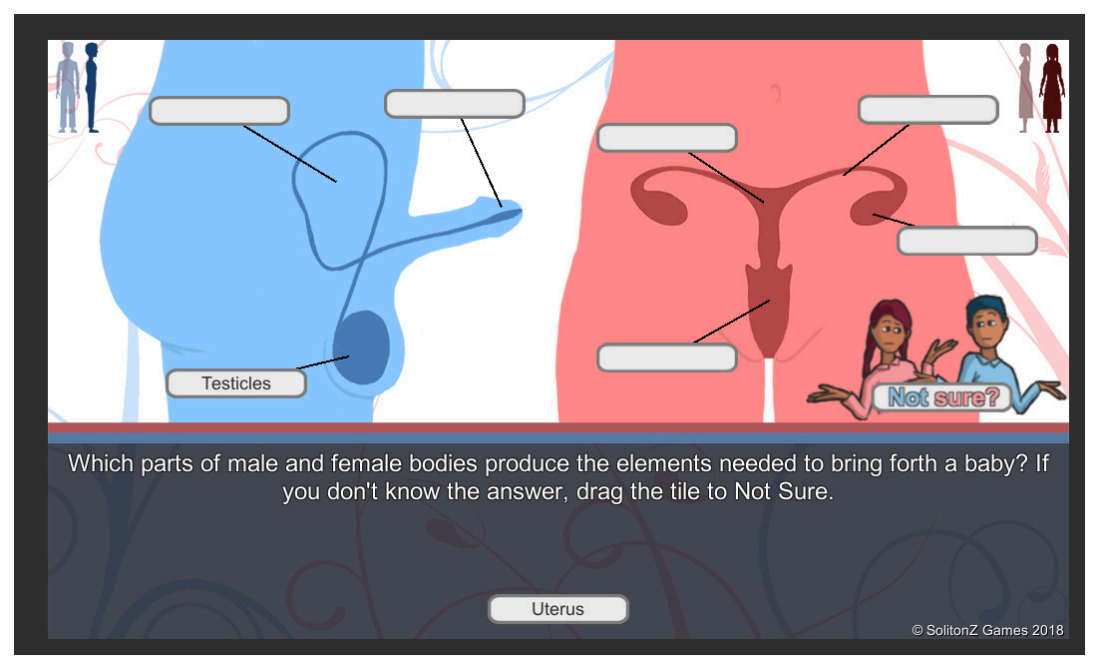
Example of pre-test anatomy question.

To assess knowledge of these topics while training students on a drag-and-drop interface, the pre-post test was designed to show male and female figures in outline, with internal organs visible. A series of 14 anatomy questions covering the full scope of in-game content was presented in a sequence of views. Players answered questions in the pre-test by dragging a word representing a concept (usually with an animation to help explain it) to the correct location on an image ( Figure 4). The structure and content of pre and post-tests is identical.

The assessment was designed to correlate with the way information is delivered in the different milestones in the game. In the MFF_2017 deployment we noted that players had a difficult time understanding where different organs were located in the body and what their functions were. In the pre-post additions, we were careful to depict both male and female bodies as a whole at the start of the pre-test. The view then zooms in to just the abdomens of the male and female bodies. We added the whole person views in the top right and left of the screen so that players could understand which view of the body was presented to them. It is very difficult to understand how organs are laid out relative to other organs. For example, in the female body, it can be difficult to show the positions of the three apertures of the urethra, vagina, and rectum relative to one another. The additional views were added to minimize this confusion.

**Figure 5.**
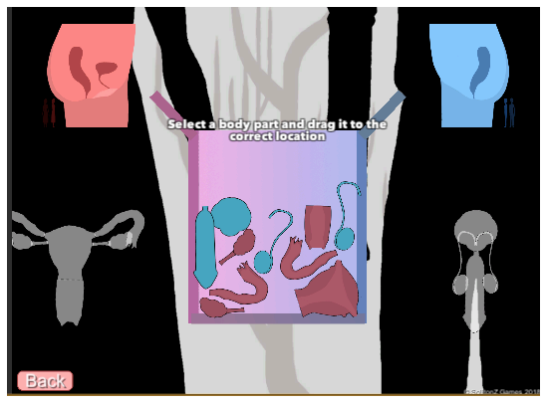
Screenshot of anatomy minigame.

Our hope was that the layout of the pre-test prior to play would prepare players to approach the anatomy section of the game where they have to drag and drop each body part to its correct location ( Figure 5). During the MFF_2017 deployment of the game, it was clear that some players did not understand the difference between the front and side views of the anatomical drawings. In addition to adding the side views in the upper right and left corners of the pre-test, we also incorporated them into the minigame. These views update as each organ is dragged into the correct location in the front view.

Solution: The drag and drop training in the pre-test was successful. Players learned how to use it in the pre-test and used it in the game without the difficulties encountered in the previous deployment.

### Pretest Challenge Three : Ensuring Fun and Making Assessment Invisible

During the early stages of the MFF_2017 deployment, teachers stayed in the room during gameplay. They often gave stern instructions on how to behave and ordered students to follow the instructions of the researchers. We realized that this made it impossible for students to experience playing the game as play. Due to the presence of their teachers, students experienced the session like a test that they were required to engage in. To encourage a sense of play, the research protocol was modified early in the first deployment to ask instructors to leave the room during gameplay. Additionally, language was added to the introductory scripts, encouraging students to play the MFF game as a game – they should only do the parts of it that they wanted to, and could stop playing at any time. This protocol was extended into the MFF_2018 deployment where considerable care was taken to encourage a sense of play and remove the pressure associated with the school environment. By introducing a pre-test, however, we recreated the circumstances under which the experience of play was potentially undermined. Students were invited into the room to play a game. However, after they are welcomed to the game, they are presented with an assessment. The 2018 deployment team reported that some students were concerned that they did not have the “right” answer and wanted to be able to go back and correct their previous answers during the pre-test. Given that the Indian system of education heavily relies on test scores and impactfully rewards those who test well, these students appeared very motivated to “do well” as soon as they realized it was an assessment.

Solution: To counter this, the researchers repeatedly stated that they should just answer what they knew and then go on to the game, but this clearly affected the experience. We learned that in future deployments, we need to add more context and less pressure to the pre-test to ensure players understand that they will not be criticized or penalized for not knowing the answers. We can actually leverage their desire to do well by encouraging them to see if they can figure out what they didn’t know in the pre-test during subsequent gameplay.

### Post-test Challenge: Adherence

We encountered more serious issues with the post-test. While the transferal of the post-test questionnaire into a digital framework did allow for personalized tracking of results, there were challenges to collecting post-game information. Qualitative feedback from the 2018 deployment team indicated that, when students came to the end of the game, and saw the same screen they had seen earlier for the pre-test, many simply dragged the tiles to “Not Sure” because it was the fastest way to get to the final screen. Others simply put down the tablet which meant that researchers had to exit the player from that game session (with no responses to the post-test questions) to reset the tablet for the next group of students. Due to the fact that we do not know exactly what happened in all the cases where there appear to be random answers to the post-test, we cannot determine how many students actually answered the questions intentionally.

Solution: Because the post-test was not only clearly an assessment, but also not well-integrated into the play experience, many players simply abandoned it. We failed to provide players with a compelling reason to want to engage in the final assessment as part of gameplay, which will be corrected in the next deployment.

## Analysis

During gameplay, tablets kept timestamped records of every user input. Data on pre- and post- test responses were saved in .csv format for statistical analysis. These datasets include information on the id number of the tablet used, the school in which the game was deployed, the self-reported sex of the player, and a unique user id for each run-through of the game. The pre-post data contains no other personalized student data.

All analyses were run in R version 3.6.0 ( Bertozzi-Villa, 2020). Overall pre- and post- test scores, as well as the percent of students who responded correctly to each question, were calculated from individual responses. On the post-test, players who responded “not sure” to every question were logged as having a “null” post-test. Score differences between groups were assessed via two-sample t-tests, and pre- to post-test score changes were assessed via one-sample t-tests.

## Ethics and consent

The study design was approved by the Institutional Review Board of the Sundaram Medical Foundation, Dr. Rangarajan Memorial Hospital, Chennai, India (IRB # IEC-09/1/2018).

Informed verbal consent was obtained from the principals of the three participating schools following consultation and a gameplay demonstration with each one. Consent was not obtained from student participants. The board deemed oral consent would suffice for the principals, and as the game covers topics which are part of the curriculum, participants’ consent was not needed. As noted earlier, once in the room students could play or not play as they wished and could put down the tablet and stop playing at any time.

## Results

The goal of this analysis was to test if embedding the game within a pre-post assessment would accurately assess how much players had learned over the course of the game. Results demonstrated that the pre-game assessment effectively captured players’ existing knowledge of human sexual anatomy and functions. The assessment tool is very helpful in demonstrating which schools are doing better with their sex ed curriculum and specifically which topics are better understood. The post-test was not effective because many players skipped it as it was not perceived as being an integral part of the experience. Lessons learned from the deployment will guide future revisions.

### Assessment of pre-test scores

**Figure 6.**
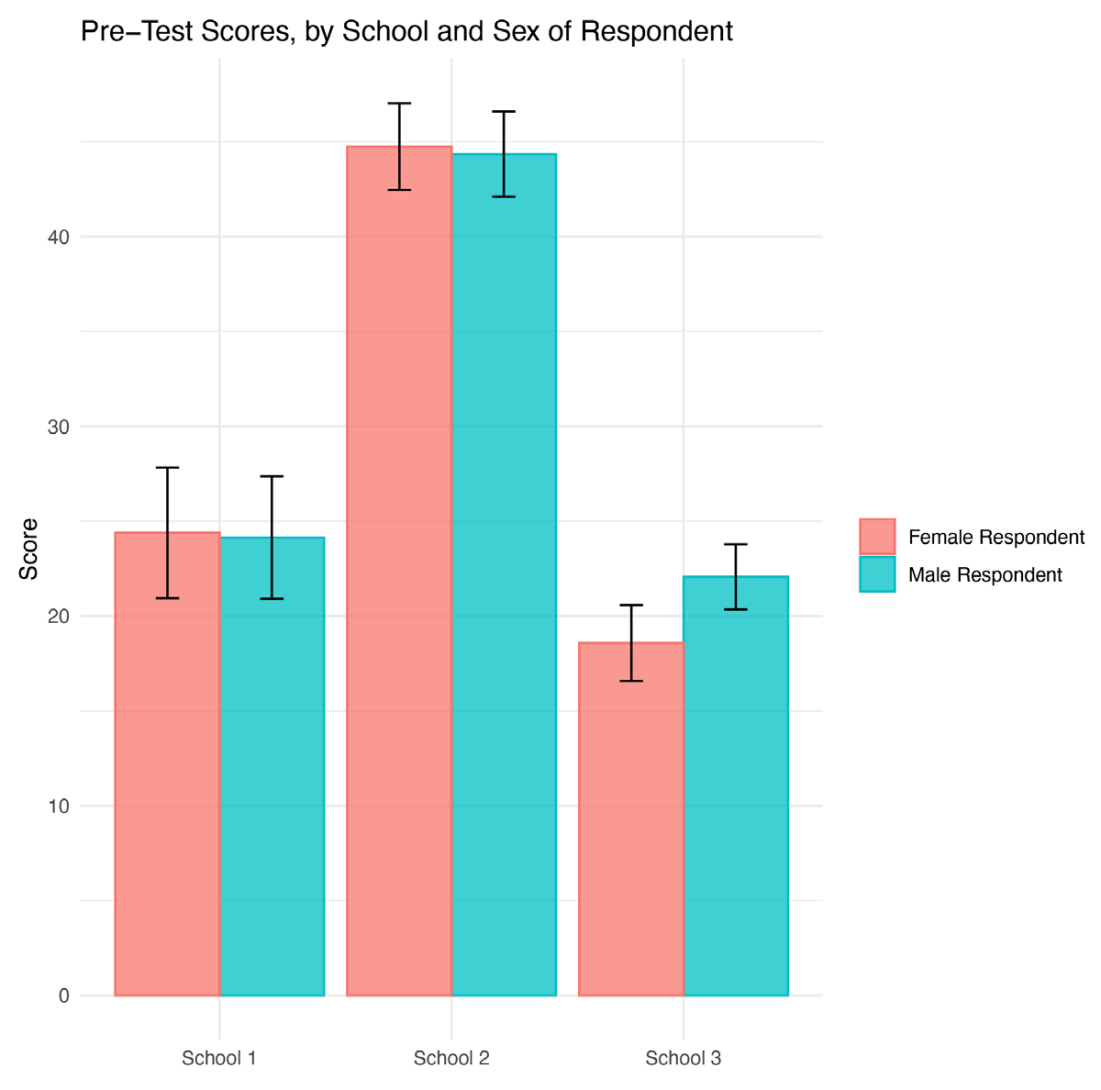
Pre-test scores by school and sex of respondent. Bars represent the standard error of the mean. N=419.

The pre- and post-test were identical, each consisting of 14 anatomy questions presented via a drag-and-drop interface with figures. Students received a single point for each question they answered correctly, and no points for incorrect responses. In the results below, test scores are always presented as percentages. A total of 419 students in three schools completed the pre-test and main game. The schools were selected based on scheduling availability and willingness to participate. The researchers from the IAPA had previously worked with these schools on AIDS education initiatives. Across all schools, the pre-test score was 33.5% on average (SE 1.15%), with substantial variation between schools. In particular, students at School 2 (who had sexual education as a formal part of their curriculum) performed significantly better than students at Schools 1 or 3 (two-tailed t-test *p<0.001*). Pre-test scores were not significantly different between male and female students at any school ( Figure 6).

**Figure 7.**
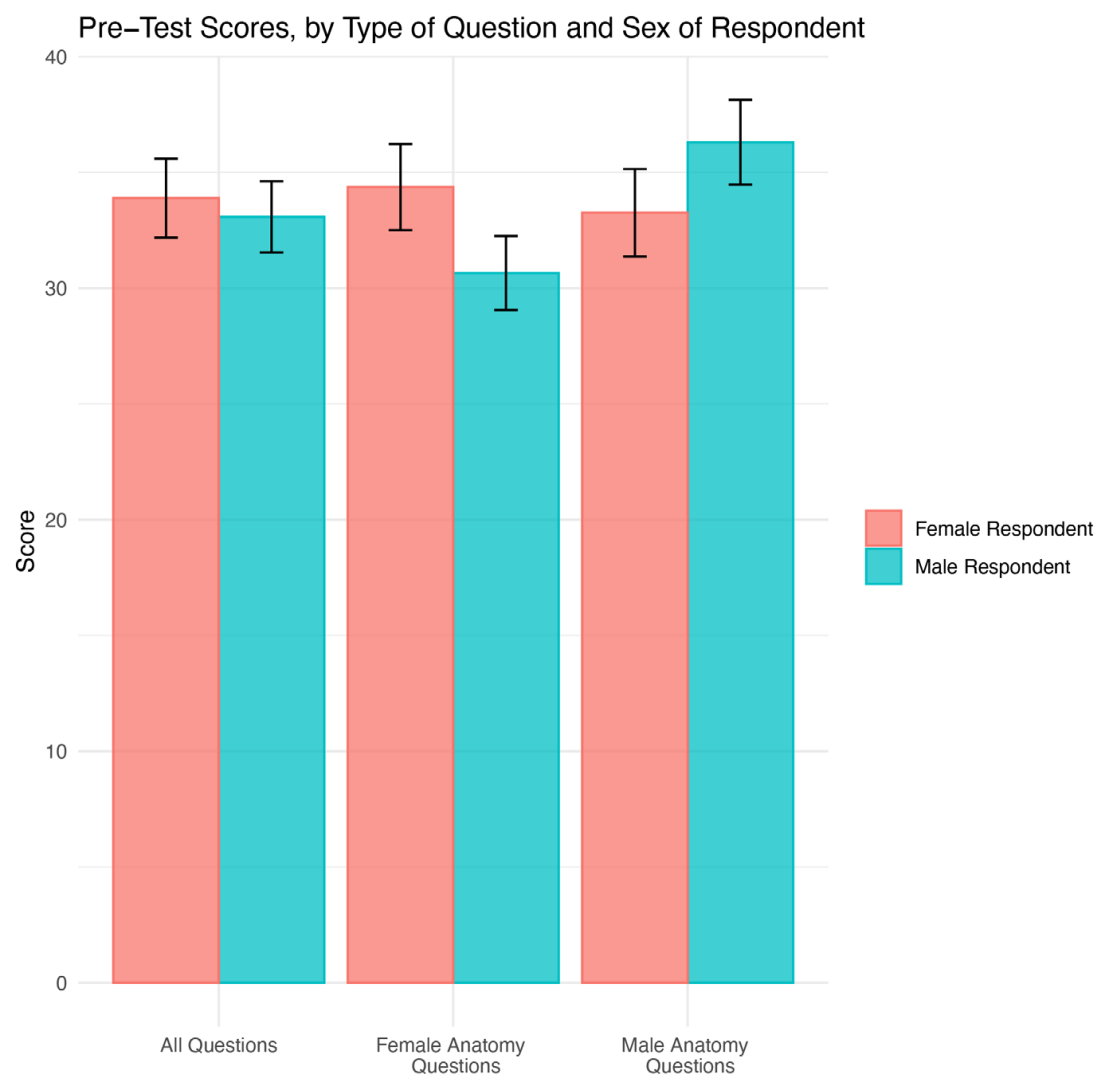
Pre-test scores by type of question and sex of respondent. Bars represent the standard error of the mean. N=419.

Across all schools, students scored slightly better on pre-test questions relating to the anatomy of their own sex compared to the opposite sex, but this effect was not statistically significant ( Figure 7). For female anatomy questions, 34.4% (SE 1.86%) of female respondents answered correctly, compared to 30.6% (SE 1.6%) of male respondents (two-tailed t-test *p=0.13*). For male anatomy questions, 33.3% (SE 1.89%) of female respondents answered correctly, compared to 36.3% (SE 1.8%) of male respondents (two-tailed t-test *p=0.25*).

**Figure 8.**
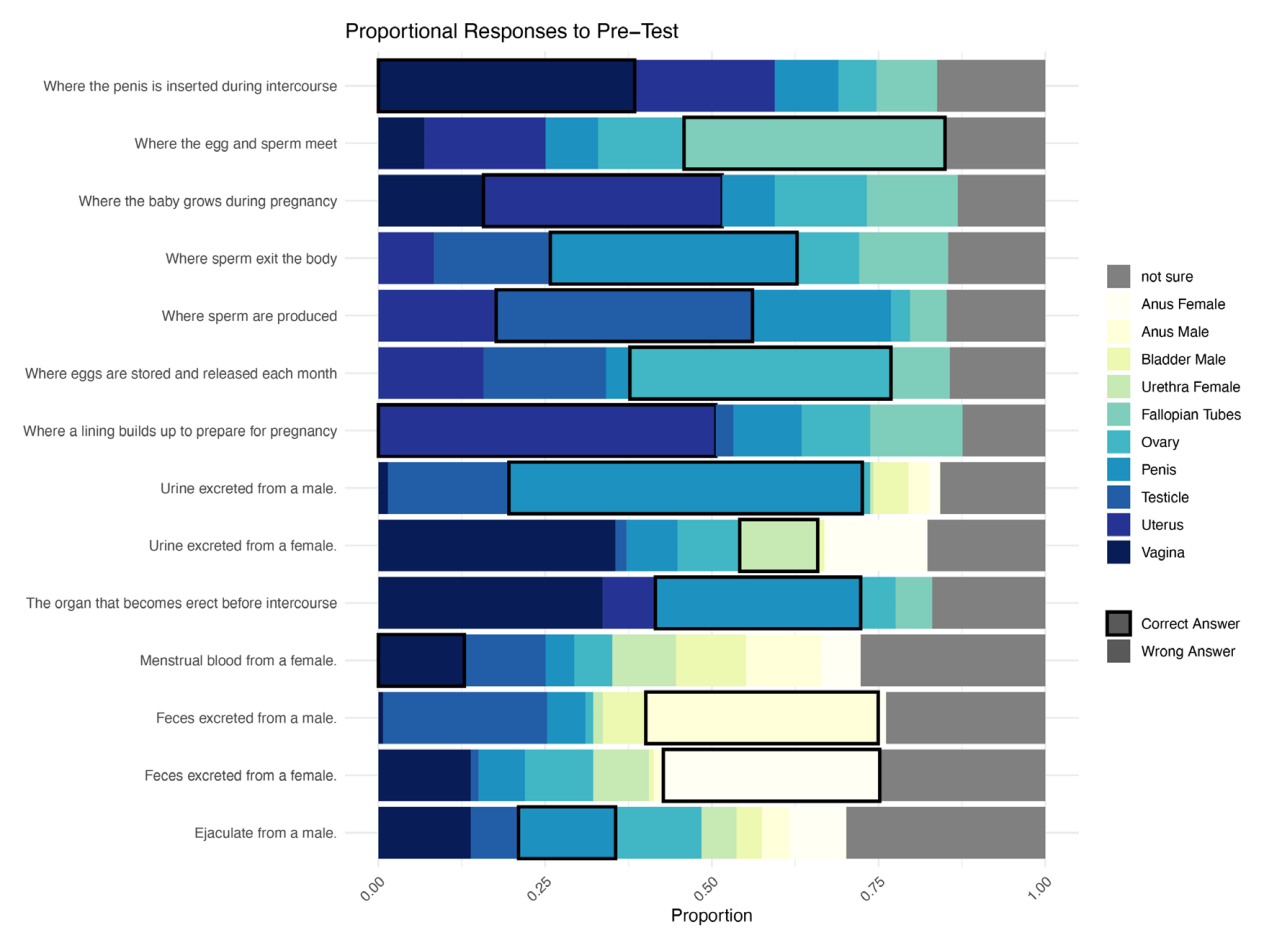
Pre-test responses by question. The highlighted bar shows the correct answer in every instance. N=419.

As shown in Figure 8, the only questions for which a majority of responses were correct were “Where is urine excreted from a male?” (53.0% correct) and “Where does a lining build up to prepare for pregnancy?” (50.6% correct). For eight of the remaining 12 questions, the correct answer received a plurality of responses, but not a majority. The four questions for which the most frequent response was not the correct answer were “Where sperm exit the body?” (plurality answer “Not Sure”, 29.8%), “where menstrual blood is excreted?” (plurality answer “Not Sure”, 27.7%), “The organ that becomes erect before intercourse?” (plurality answer “Vagina”, 33.7%), and “Where urine is excreted from a female? (plurality answer “Vagina”, 35.6%)”.

### Pre-post test assessment

**Figure 9.**
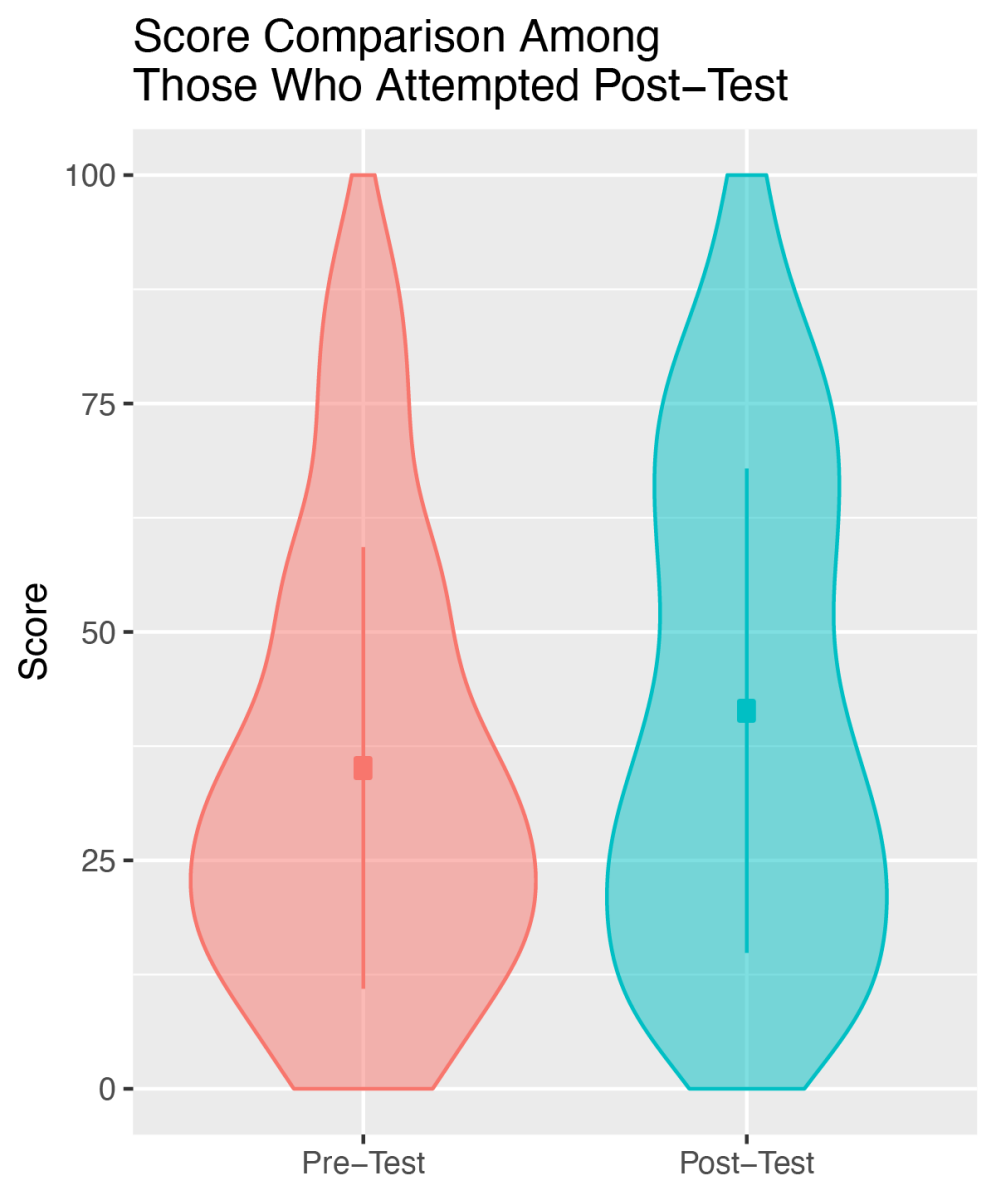
Violin plot of score distributions in the pre- and post-test, for those students who completed both tests (N=246). Points represent means, and bars represent two times the standard error.

As described above, assessment of knowledge gain was complicated by the large number of students who did not complete the post-test or who rushed through it, answering “not sure” to all questions (173 students, 41.3% of total). We refer to this group as having a “null” post-test. While it is not possible to assess knowledge gain among those with a null post-test, among the 246 (58.7%) students who did attempt the post-test we find on average a 6.27-percentage-point score gain between pre- and post- tests (95% CI 3.8–8.75, *p<0.001,* one-sample t test, Figure 9). Scores ranged from 0 to 100 for both tests. In the pre-test, 17 students answered all questions incorrectly while one student answered perfectly. In the post-test, five students answered all questions incorrectly and five students answered perfectly.

**Figure 10.**
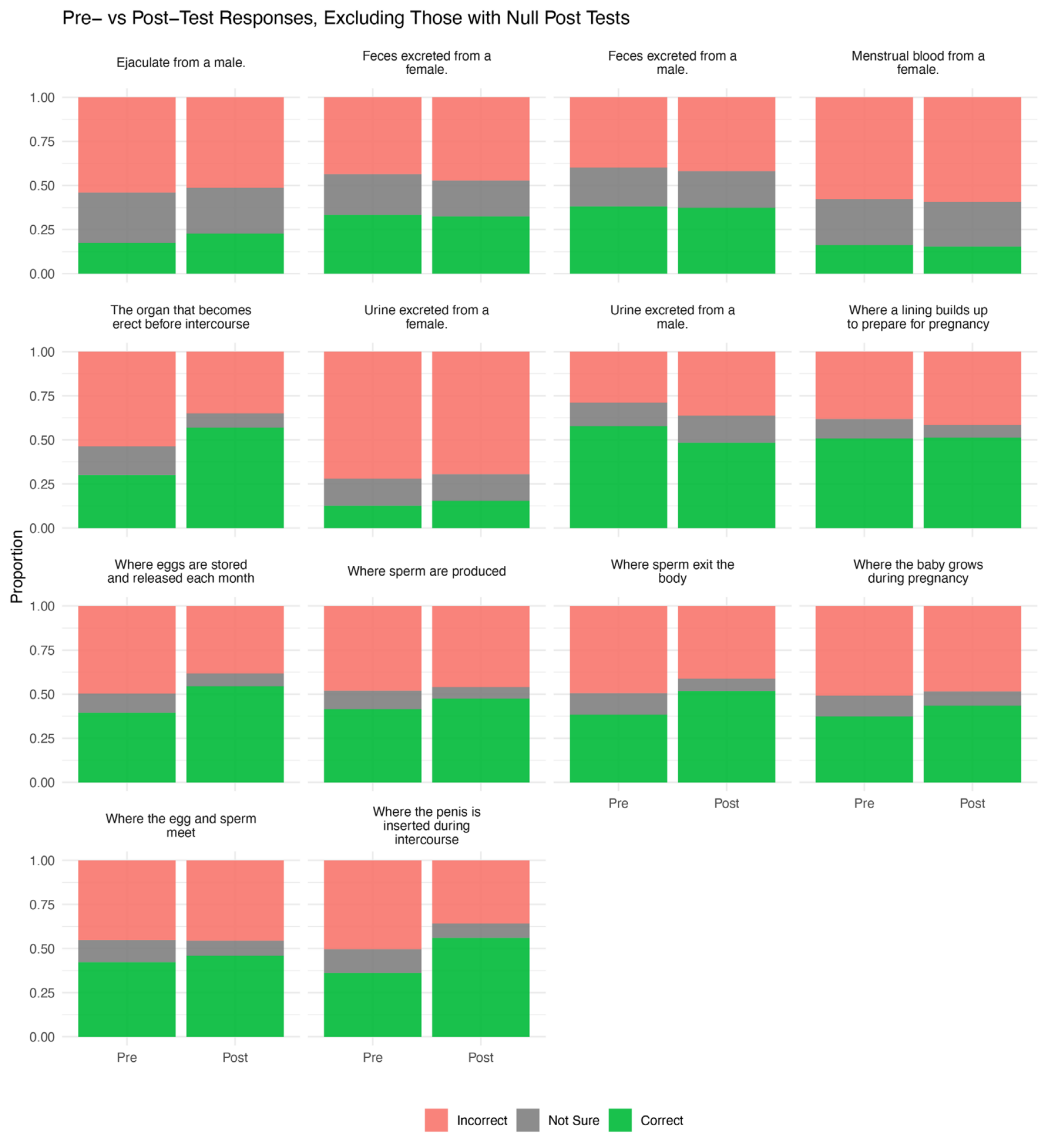
Question-by-question comparison of pre- and post- test responses, by proportion, excluding those with “null” post-tests (N=246).

A question-by-question breakdown of pre- vs post- test result among those who attempted the post-test shows the largest knowledge gain around topics of intercourse, egg storage, and sperm movement ( Figure 10).

## Discussion

Initial analysis of the results suggested that there was little knowledge gain as a result of gameplay. There was very little change between pre- and post-test results on many of the metrics. Discussions with the deployment teams and more detailed analysis of the results produced a more nuanced understanding of what happened. Many players simply did not complete the post-test or randomly swiped to finish as quickly as possible. When the data for players who did complete both the pre- and post-tests with intention was analyzed separately, there was a modest but notable increase in knowledge. Additionally, we determined that the pre-test was a useful tool for assessing prior knowledge and therefore the efficacy of sexual education programs at different schools.

We learned a great deal about the difficulty of creating effective pre-post assessments for a game that includes sensitive topics. Adolescents offered a game of this type are already nervous and excited about it. The process of setting up a context in which their current knowledge is assessed needs to be approached carefully. We encountered several pitfalls that complicated the assessment process and which affected the validity of the assessment data. We are able to conclude that using a game to assess current knowledge of reproductive anatomy and processes can be very effective. In order to assess knowledge gain after gameplay, students need to be motivated to fully engage in the post-test assessment. For future deployments of the game, we plan to change the deployment protocol to address the issues discussed in this report and better integrate the pre-post testing process in the overall experience.

Other researchers have addressed the problem of lack of participation in post-testing in creative ways. Chittaro & Buttusi (2020) included a pre-post test in their widely distributed aircraft cabin safety game. In addition to the data collected from players who completed the pre-post, they collected data about the kinds and frequency of errors that players made while playing the game and included that analysis in their reporting on efficacy and knowledge gain. There are two minigames in the MFF game where players are dragging and dropping objects to learn how different reproductive systems work. In future deployments, we can track how often objects are misplaced or dropped and integrate these metrics into our efficacy assessment. Additionally, we can track how often players replayed minigames and how this correlates to performance on pre-post tests. In their meta-analysis of digital learning through games, Clark *et al.* (2016) suggest several ways to enhance leaning and assessment including: allowing players to have unlimited access to the game, embedding the game within existing classroom assessment measures, and including supplemental non-game instruction. None of these options were available to us in the India deployments, but they can guide our attempts to distribute the game in the future.

Other ways to more accurately assess the impact of the game include expanding the scope of assessment to include more qualitative metrics. Other researchers have noted that playing games about sensitive topics can result in players feeling more comfortable about the topic and reducing stigma ( Arnab *et al.*, 2013; Ferchaud *et al.*, 2020). The deployment teams during both deployments noted that prior to playing the game, the atmosphere in the room was somewhat tense, but that after everyone had played the game players were relaxed and laughing and appeared more comfortable asking questions about reproduction and sexuality. In future deployments, we will seek to measure this impact and include self-efficacy and affective outcomes as described in several of the studies reported by ( Boyle *et al.*, 2016)

It is standard practice in serious game development to seamlessly integrate assessment into the existing structure of the game ( Klopfer *et al.*, 2018; Serrano-Laguna *et al.*, 2018). As we have shown, this is difficult in a game that deals with a sensitive topic. Our plan going forward is to address this challenge openly in the introduction to the game experience. After players open the tablet, we will have an animated character appear who discusses the fact that what will follow is a game about sexuality and that this is a difficult topic for many people to talk about. After normalizing the idea of embarrassment, the character will then introduce the idea that knowledge is power and that the game will help players learn about things that are important to their future. Then the pre-post will be presented as a challenge…” let’s see how much you know now and then see if after you play the game you know all the answers to things you didn’t know before.” Hopefully, with this context, we will avoid the pitfalls of our Chennai deployment.

## Conclusion

This deployment demonstrated that a game-based tool can be an effective means of gathering information. We learned that many adolescents in these schools lack basic knowledge of human anatomy and sexuality, especially given that the students chosen had already received baseline training in HIV prevention and are likely better informed than other students. We observed that in addition to collecting data and educating players, games of this type also have the potential for de-stigmatizing conversations about sex and sexuality which we will seek to quantify in future deployments. The deployment also provided us with important information for improving the tool.

## Data availability

### Underlying data

Open Science Framework: Outcomes Assessment Pitfalls: Challenges to Quantifying Knowledge Gain in a Sex Education Game. https://doi.org/10.17605/OSF.IO/WMHCD ( Bertozzi-Villa *et al.*, 2020)

This project contains the following underlying data

- prepost.csv (Questions and responses to all pre- and post- tests administered, along with timestamps and other metadata)

### Extended data

Pre- and post-test data were analyzed and visualized using R version 3.6.0. All code is available from GitHub ( https://github.com/bertozzivill/india-family-planning) and archived with Zenodo ( http://doi.org/10.5281/zenodo.3822455 ( Bertozzi-Villa, 2020))

Data are available under the terms of the Creative Commons Zero "No rights reserved" data waiver (CC0 1.0 Public domain dedication).

## Software availability

An installable and playable version of the game and all data used for analysis is publicly available at Open Science Framework, as described below.

Archived source code at time of publication: https://doi.org/10.17605/OSF.IO/WMHCD ( Bertozzi-Villa *et al.*, 2020)

License: MIT

## Notes

^i^The project was funded by a Grand Challenges in Global Health grant ( https://gcgh.grandchallenges.org/grant/childbearing-intentions-and-family-planning-game).

^ii^Due to cultural taboos in India which would have made it impossible to deploy the game at all, same-sex marriage was not an option in the game.
