# Supplementary material for: Outcomes assessment pitfalls: challenges to quantifying knowledge gain in a sex education game
Source: Gates Open Res. 2021 Feb 16;4:73. Originally published 2020 Jun 30. [Version 3] doi: 10.12688/gatesopenres.13129.3 (PMC7993112; doi:10.12688/gatesopenres.13129.3)
Supplement: Peer review history

# Peer review: Reviewer response for version 3

**DOI:** 10.21956/gatesopenres.14451.r30420

**Published:** 2021-03-23

**Authors:** Fabio Buttussi

**Affiliations:** 1 Human-Computer Interaction Lab, Department of Mathematics, Computer Science and Physics, University of Udine, Udine, Italy

The authors addressed all the minor issues pointed out in the previous review and further improved their paper.

 In particular, the link to the video is very useful to understand the methodology, the literature is now considered and discussed, and the minor problems concerning presentation were fixed.

 In my opinion, the paper is ready to be approved.

Is the work clearly and accurately presented and does it cite the current literature?

No

If applicable, is the statistical analysis and its interpretation appropriate?

Yes

Are all the source data underlying the results available to ensure full reproducibility?

Yes

Is the study design appropriate and is the work technically sound?

Partly

Are the conclusions drawn adequately supported by the results?

Partly

Are sufficient details of methods and analysis provided to allow replication by others?

Partly

Reviewer Expertise:

My research interests concern human-computer interaction, virtual reality, artificial intelligence, and serious games. I explore different application domains for my research such as aviation safety, health and fitness, emergency medicine, and sing languages, with a particular focus on training.

I confirm that I have read this submission and believe that I have an appropriate level of expertise to confirm that it is of an acceptable scientific standard.

# Peer review: Reviewer response for version 2

**DOI:** 10.21956/gatesopenres.14413.r29986

**Published:** 2020-12-24

**Authors:** Fabio Buttussi

**Affiliations:** 1 Human-Computer Interaction Lab, Department of Mathematics, Computer Science and Physics, University of Udine, Udine, Italy

This article investigates the use of a serious game in a new interesting application domain, i.e. to teach adolescents about sex education. While the first version presented the research as a user evaluation that was affected by several methodological issues, the revised version successfully refocused the research as series of lessons learned from two user evaluations and the problems encountered by the authors. Some of these problems are peculiar to the chosen domain, while others could be generalized to other domains where serious games can be used. This latter aspect makes the contribution interesting also for computer science researchers as well as educators that are not working in the specific domain. Despite the new version cannot resolve the methodological issues in the design of the user evaluation (this is the motivation for my “Partly” answer to the question about technical soundness), the new focus makes those issues not critical, so I think that they will no longer prevent the acceptance of the paper.

 The main reason why I will not yet approve the article instead concerns the very limited discussion of the results against the literature. I acknowledge that the considered application domain is largely unexplored, but serious games have been used in many other domains and some of the problems encountered by the authors have already been addressed in previous work. One peculiar example I am familiar with is the problem of players not devoting proper attention to assessment tests, which was described in a recent article. ^1^ In particular, that article described the criteria applied by the authors to identify a subset of users who completed pre- and post-tests before and after playing a serious game among the millions of users who played it. Moreover, that article shows how in-game metrics could be used to assess improvement in the taught topic also among users who did not complete the pre- and post-tests, and the authors of the submitted paper or other researchers might be interested in applying such an approach to serious games for sex education in their future work. More generally, I think the article will benefit from a discussion of the results that compare authors’ findings with those that emerged from previous research on serious games (e.g., see the systematic reviews about serious games). ^2^^,^^3^^,^^4^

 While the authors provided the links to code and installers of the serious game, I think that methods could be improved by adding some more details about how the serious game works, because several readers may not be willing to install the serious game on their devices and the article provides only one example of an included mini-game. A video of the gameplay would also be very useful.

 As a minor issue, there are some problems with the terminology used in the paper. For example, pre- and post-test are called in different ways in different parts of the paper (post test, post-test, post- test, post assessment, post-game assessment). Similarly, serious games are sometimes referred to as applied games, and this can make the readers wonder if there are any differences between the two terms. It would also be useful that the authors clearly report the scale of scores of pre- and post-tests: the pre-test score is first reported as a percentage, but later the gain is reported in points, and the maximum score is reported only in Figure 9.

 Overall, I think that the revision solved almost all critical issues and a minor revision would be sufficient to discuss the results against the literature and fix some minor language or presentation issues.

Is the work clearly and accurately presented and does it cite the current literature?

No

If applicable, is the statistical analysis and its interpretation appropriate?

Yes

Are all the source data underlying the results available to ensure full reproducibility?

Yes

Is the study design appropriate and is the work technically sound?

Partly

Are the conclusions drawn adequately supported by the results?

Partly

Are sufficient details of methods and analysis provided to allow replication by others?

Partly

Reviewer Expertise:

My research interests concern human-computer interaction, virtual reality, user-adaptive and context-aware systems, mobile devices, and serious games. I explore different application domains for my research such as aviation safety, health and fitness, emergency medicine, and sing languages, with a particular focus on training.

I confirm that I have read this submission and believe that I have an appropriate level of expertise to confirm that it is of an acceptable scientific standard, however I have significant reservations, as outlined above.

## References

[1] : Learning Safety through Public Serious Games: A Study of. *IEEE Trans Vis Comput Graph*.2020;PP: 10.1109/TVCG.2020.3022340 10.1109/TVCG.2020.3022340 32894716

[2] : A systematic literature review of empirical evidence on computer games and serious games. *Computers & Education*.2012;59(2) : 10.1016/j.compedu.2012.03.004 661-686 10.1016/j.compedu.2012.03.004

[3] : An update to the systematic literature review of empirical evidence of the impacts and outcomes of computer games and serious games. *Computers & Education*.2016;94: 10.1016/j.compedu.2015.11.003 178-192 10.1016/j.compedu.2015.11.003

[4] : Digital Games, Design, and Learning: A Systematic Review and Meta-Analysis. *Rev Educ Res*.2016;86(1) : 10.3102/0034654315582065 79-122 10.3102/0034654315582065 26937054PMC4748544

# Peer review: Reviewer response for version 2

**DOI:** 10.21956/gatesopenres.14413.r29984

**Published:** 2020-12-09

**Authors:** Melissa Gilliam

**Affiliations:** 1 Department of Obstetrics and Gynecology, University of Chicago, Chicago, IL, USA

The changes are adequate. Thank you for the opportunity to review again.

Is the work clearly and accurately presented and does it cite the current literature?

No

If applicable, is the statistical analysis and its interpretation appropriate?

Yes

Are all the source data underlying the results available to ensure full reproducibility?

Yes

Is the study design appropriate and is the work technically sound?

Partly

Are the conclusions drawn adequately supported by the results?

Yes

Are sufficient details of methods and analysis provided to allow replication by others?

Partly

Reviewer Expertise:

ASRH, game-based learning

I confirm that I have read this submission and believe that I have an appropriate level of expertise to confirm that it is of an acceptable scientific standard.

# Peer review: Reviewer response for version 1

**DOI:** 10.21956/gatesopenres.14316.r29409

**Published:** 2020-09-15

**Authors:** Claudia Regina Furquim de Andrade

**Affiliations:** 1 Department of Physiotherapy, Speech Therapy and Occupational Therapy, Faculty of Medicine FMUSP, University of Sao Paulo, Sao Paulo, Brazil

I would like to thank you for the opportunity to review the manuscript entitled “Outcomes assessment pitfalls: challenges to quantifying knowledge gain in a sex education game” that has been submitted to Gates Open Research.

 The study investigated and discussed the challenges associated with incorporating educational assessment before and after the use of an educational game entitled 'My Future Family Game', delivered to students of three schools in and around Chennai, India. The authors investigated the efficacy of a pre- and post-test assessment. I consider this topic relevant and interesting, and in need of further investigation, especially with the application of experimental designs. Therefore, the authors are commended for the novel and trendy approach. However, I note several methodological concerns. Among these, relevant literature regarding the use of educational games, particularly concerning outcomes assessment, is not reviewed (and was not used to guide the research design); inclusion and exclusion criteria, for both participants and schools, are not specified and may not have been controlled; and the study methods are not described in sufficient detail. For these reasons, I am concerned that it is not possible to fairly review the results and conclusions reached. Furthermore, I would suggest publishing this manuscript as a research note, since it mostly describes the unexpected observations and lab protocols.

 Comments on the Introduction:

- In general, the background/rationale for this study is not sufficiently developed as written. The study is not hypothesis-driven and predicted outcomes are not provided.
- The introduction, albeit interesting, is essentially a description of the game used for this study, detailing its design and development.

 

 Comments on the Methods:

- I believe most of the information concerning the game development and pilot studies could be presented, in short, in the methods section, with citations to the previous published studies. In addition, the methods section should clearly and succinctly explain the study methods, so that it can be duplicated.

- Inclusion and exclusion criteria, for both participants and schools, are not provided (for example, were the schools chosen only by convenience? What about the student participants in each school, how were they selected?). Demographic information about included participants is not very substantial either. The design of the pre- and post-test assessment is also not clearly described.

 

 Comments on the Results:

- The manuscript depicts a lack of interest from many participants in completing the educational assessments. I suggest that the authors elaborate on what they believe was causing this lack of interest. Could it be influencing the results?

 

 Comments on the Discussion:

- The manuscript would benefit if the authors discussed their findings on relevant literature. The authors did not cite and engage with the pertinent literature regarding educational assessment.

Is the work clearly and accurately presented and does it cite the current literature?

No

If applicable, is the statistical analysis and its interpretation appropriate?

Yes

Are all the source data underlying the results available to ensure full reproducibility?

Yes

Is the study design appropriate and is the work technically sound?

Partly

Are the conclusions drawn adequately supported by the results?

Yes

Are sufficient details of methods and analysis provided to allow replication by others?

No

Reviewer Expertise:

I am a Full Professor at the Faculty of Medicine at the University of São Paulo, and published 155 articles in indexed journals and 376 papers in the annals of events. I have additional 96 publications, between book chapters and books, and 361 items of technical production. Between 1989 and 2020 I participated in 24 research projects, 22 of which I coordinated. I am also the coordinator of three specialization courses.

I confirm that I have read this submission and believe that I have an appropriate level of expertise to state that I do not consider it to be of an acceptable scientific standard, for reasons outlined above.

# Response

**Published:** 2020-10-28

**Authors:** Elena Bertozzi

**Affiliations:** Quinnipiac University, USA

Thank you very much for the detailed and thorough feedback in this review. We have implemented your suggestions as detailed below.

 1.     Added literature review.

 2.     Added inclusion and exclusion criteria.

 3.     The study is not hypothesis driven. The paper seeks to add to the scarce literature in the field relating to assessment of the efficacy of serious games to educate players about sexual anatomy and reproduction.

 4.     The introduction and other sections of the paper have been revised to clarify the paper’s purpose and arguments.

 5.     The results section was edited to clarify the reasons for the lack of participation in the post test and add suggestions to resolve the issues we encountered.

 6.     The discussion section was edited to clarify connections to the pertinent literature.

 The paper was reorganized to clarify the purpose of the study and how publication of the issues we met and resolved could be useful to other researchers.

# Peer review: Reviewer response for version 1

**DOI:** 10.21956/gatesopenres.14316.r29297

**Published:** 2020-08-17

**Authors:** Melissa Gilliam

**Affiliations:** 1 Department of Obstetrics and Gynecology, University of Chicago, Chicago, IL, USA

Thank you very much for the opportunity to review this paper. In it, the authors consider how to integrate knowledge assessment into a sexual health game intervention entitled 'My Future Family Game' delivered to young people in Chennai, India.

 This game-based intervention aims to collect information about family planning intentions. Here, the authors consider the efficacy of a pre-post assessment and the post-test assessment in particular. They describe the development process and some of the pitfalls of the approach they took.

 Overall, the paper is interesting and I enjoyed reading about the logistics and decision making that the team underwent. Working internationally on game development can be difficult and it was interesting to see how the team tackled field research. That said, this seems to be a report about processes and decisions that were made rather than a generalizable paper couched in the larger literature.

**Specific points:**

**Introduction:**

- If this paper is about game assessment, then the introduction should provide a literature review on that topic.
- The introduction turns into a description of pilot game design and development. Instead, this information perhaps belongs in the methods section.

**Methods:**

- The methods section should be the planned study or evaluation. Instead it seems almost like a results section in that you state how many people were involved etc.
- The section on challenges from first deployment again seems like the result section. Regardless, it is a bit confusing as to how this relates to an assessment of knowledge.

**Results:**

- It seems that the many pitfalls are avoidable and others have successfully done game-based assessment. It is not clear whether these are particular to the ways the researchers set up the assessment or inherent to assessment, I would argue for the former.

**Discussion:**

- Statements such as “our response to the results was dismay” is quite casual and instead perhaps the discussion could be used to review the literature on game assessment.
- It is not clear that pre post assessment issue is particular to sensitive topics and can arise for other reasons

 

Is the work clearly and accurately presented and does it cite the current literature?

No

If applicable, is the statistical analysis and its interpretation appropriate?

Yes

Are all the source data underlying the results available to ensure full reproducibility?

Yes

Is the study design appropriate and is the work technically sound?

Partly

Are the conclusions drawn adequately supported by the results?

Yes

Are sufficient details of methods and analysis provided to allow replication by others?

Partly

Reviewer Expertise:

ASRH, game-based learning

I confirm that I have read this submission and believe that I have an appropriate level of expertise to state that I do not consider it to be of an acceptable scientific standard, for reasons outlined above.

# Response

**Published:** 2020-10-28

**Authors:** Elena Bertozzi

**Affiliations:** Quinnipiac University, USA

Thank you for this thoughtful review and for the suggestions that guided our revisions to the paper.

 Specific Points:

 Intro: We added a literature review and analysis of results which we used to focus the introduction of the paper. The introduction now clarifies that the topic of the paper is refining the process of outcomes assessment for a serious game, specifically one that addresses sexual anatomy and reproduction.

 Methods: We reorganized the paper so that the methods section explains the process that we undertook based on the first and second deployments of the game and problems associated with the addition of the pre/post assessment.

 Results: There is a growing body of research on game-based assessment, however there is little published work in the area of games related to education about sex and reproduction. The assessment of knowledge gain without in person interviews is complicated by numerous factors and our paper seeks to provide guidance in this evolving area.

 Discussion: We have revised this section to remove unprofessional language and clarify the connections between the assessments and issues relating to serious game deployments.

 The paper has been reorganized for clarity.
